# Supplementary material for: Anaerobic riboflavin degradation by human gut Lachnospiraceae
Source: bioRxiv. 2026 Feb 16:2026.02.13.705874. Preprint. [Version 1] doi: 10.64898/2026.02.13.705874 (PMC12934955; doi:10.64898/2026.02.13.705874)
Supplement: Supplement 1 [file NIHPP2026.02.13.705874v1-supplement-1.pdf]

bioRxiv preprint doi: https://doi.org/10.64898/2026.02.13.705874; this version posted February 18, 2026. The copyright holder for this preprint (which was not certified by peer review) is the author/funder, who has granted bioRxiv a license to display the preprint in perpetuity. It is made available under aCC-BY 4.0 International license.

Supplementary Information

Supplementary Table S1: Detailed strain information for all strains used in this study.

Supplementary Table S2: Table of all KOfams present in *D. formicigenerans*, *R. peoriensis,* and *A. caccae* but not *C. scindens* and *B. coccoides.*

Supplementary Table S3: Table of all KOfams present in *C. scindens* and *B. coccoides* but not *D. formicigenerans, R. peoriensis,* or *A. caccae*.

Supplementary Table S4: *Lachnospiraceae* representative genomes used for calling orthogroups.

Supplementary Table S5: Table of all potentially orthologous *fin* neighborhoods detected.

Supplementary Table S6: Bacteroidales representative genomes used for calling orthogroups.

Supplementary Figure S1: Read coverage plots across the *fin* neighborhood. A) 17.5μM riboflavin, 11 hours. B) 0.13μM riboflavin, 12 hours.

Supplementary Figure S2: Orthogroup gene trees for three genes in the fin neighborhood: A) bifunctional riboflavin kinase/FAD synthetase (*finB*), B) aldolase (*finC*), and C) aldehyde dehydrogenase (*finG*). The *C. scindens* and *F. fissicatena* orthologs are labeled, and the clades selected for identifying potential orthologous neighborhoods are highlighted in yellow.

Supplementary Figure S3: Diagram showing detected *fin* gene neighborhoods in *C. scindens, M. intestini, F. fissicatena,* and *M. hominis.* Genes in the same orthogroup as a *C. scindens fin* gene are in pink; flanking genes that had a Prokka annotation (other than “hypothetical gene”) are labeled with their inferred function.
